# Association of Symptoms After COVID-19 Vaccination With Anti–SARS-CoV-2 Antibody Response in the Framingham Heart Study

**DOI:** 10.1001/jamanetworkopen.2022.37908

**Published:** 2022-10-21

**Authors:** Emilia A. Hermann, Benjamin Lee, Pallavi P. Balte, Vanessa Xanthakis, Beth D. Kirkpatrick, Mary Cushman, Elizabeth Oelsner

**Affiliations:** 1 Division of General Medicine, Department of Medicine, Columbia University, New York, New York; 2 Department of Pediatrics, Larner College of Medicine at the University of Vermont, Burlington; 3 Department of Medicine, Boston University School of Medicine, Boston, Massachusetts; 4 Department of Biostatistics, Boston University School of Public Health, Boston, Massachusetts; 5 Department of Medicine, Larner College of Medicine at the University of Vermont, Burlington; 6 Department of Microbiology and Molecular Genetics, Larner College of Medicine, University of Vermont, Burlington

## Abstract

This cohort study examines the association of self-reported postvaccination symptoms with anti–SARS-CoV-2 antibody response among Framingham Heart Study participants contributing to the Collaborative Cohort of Cohorts for COVID-19 Research study.

## Introduction

SARS-CoV-2 messenger RNA (mRNA) vaccines (BNT162b2 [Pfizer-BioNTech] and mRNA-1273 [Moderna]) are associated with local and systemic symptoms; however, whether postvaccination symptoms are associated with vaccine-induced antibody response is unknown. Previous studies^1,2,3^ of COVID-19 vaccine reactogenicity and immunogenicity were limited to convenience samples that may not be generalizable. We studied the association of self-reported postvaccination symptoms with anti–SARS-CoV-2 antibody response among Framingham Heart Study (FHS) participants contributing to the Collaborative Cohort of Cohorts for COVID-19 Research (C4R) study.^4^

## Methods

The FHS is an ongoing, prospective cohort study evaluating cardiovascular disease risk factors. In February 2021, participants were invited to self-administer C4R questions on COVID-19 vaccination (and associated symptoms) and submit a dried blood spot to test for anti–SARS-CoV-2 antibodies (eFigure in the Supplement). This report includes participants who received 2 doses of mRNA vaccine at least 2 weeks before blood spot collection. Postvaccination symptoms were categorized as systemic symptoms (fever, chills, muscle pain, nausea, vomiting, headache, and/or moderate to severe fatigue) or local symptoms (injection site pain and/or rash). IgG antibodies to SARS-CoV-2 spike subunit were measured using microsphere immunoassay (Luminex), chosen for its successful use in population-based serosurveys. Results were reported as median fluorescence intensity (MFI), with batch-specific reactive antibody response MFI cutoffs.^5^ Associations between postvaccination symptoms and antibody response were assessed by χ^2^ test and multivariable linear regression, with complete case analyses adjusted for batch, time since vaccination, and sociodemographic and clinical characteristics. A 2-sided *P* < .05 was considered statistically significant. Protocols were approved by institutional review boards of participating institutions and the National Heart, Lung, and Blood Institute. Written informed consent was obtained from all participants. This study followed the Strengthening the Reporting of Observational Studies in Epidemiology (STROBE) reporting guideline.

## Results

Of 3200 FHS participants eligible to participate in C4R, 928 (29%) completed the C4R questionnaire and blood spot collection and reported 2 doses of BNT162b2 (414 [45%]) or mRNA-1273 (514 [55%]) vaccines (eFigure in the Supplement). Respondents’ mean (SD) age was 65 (12) years, 360 (39%) were men and 568 (61%) were women, 893 (96%) were non-Hispanic White, and 84 (9%) self-reported prior COVID-19 infection. After either vaccine dose, 446 participants (48%) reported systemic symptoms, 109 (12%) reported local symptoms only, and 373 (40%) reported no symptoms. In bivariate analysis, symptoms were associated with younger age, female sex, prior infection, and the mRNA-1273 vaccine (Table). Antibody reactivity was observed in 365 asymptomatic participants (98%), 108 participants (99%) with only local symptoms, and 444 participants (99%) with systemic symptoms (*P* = .08). In adjusted models, systemic symptoms were associated with greater antibody response, although associations were attenuated with sequential adjustment for potential confounders (Figure). Similar results were obtained with exclusion of participants with prior COVID-19 infection.

**Table.** Characteristics of Study Participants by Self-reported Symptoms After SARS-CoV-2 Messenger RNA Vaccination^a^

Characteristic	No symptoms (n = 373)	Local symptoms only (n = 109)	Systemic symptoms (n = 446)	*P* value^b^
Age, mean (SD), y	68 (12)	69 (11)	62 (12)	<.001
Sex				
Male	195 (52)	38 (35)	127 (28)	<.001
Female	178 (48)	71 (65)	319 (72)	
Race and ethnicity				
White	362 (97)	105 (96)	426 (95)	.40
Other racial or ethnic group^c^	10 (3)	4 (4)	20 (5)	
Missing	1 (0)	0	0	
Vaccine				
BNT162b2	214 (57)	41 (38)	159 (36)	<.001
mRNA-1273	159 (43)	68 (62)	287 (64)	
BMI, mean (SD)	28 (5)	28 (6)	28 (6)	.87
Smoking status				
Never	191 (51)	53 (49)	242 (54)	.72
Former	146 (39)	46 (42)	170 (38)	
Current	36 (10)	10 (9)	34 (8)	
Comorbidities				
Hypertension	159 (43)	45 (42)	163 (37)	.21
Diabetes	39 (10)	16 (15)	47 (11)	.41
Coronary heart disease	32 (9)	9 (8)	31 (7)	.67
Heart failure	4 (1)	3 (3)	1 (0)	.03
eGFR >60 mL/min/1.73 m^2^	344 (93)	99 (93)	425 (96)	.26
Stroke or TIA	12 (3)	3 (3)	8 (2)	.42
Prior COVID-19	19 (5)	6 (6)	59 (13)	<.001
MFI S-antibody reactive	365 (98)	108 (99)	444 (99)	.08
Log-antibody, mean (SD)	8 (1)	9 (1)	9 (1)	<.001
Time from vaccination, mean (SD), d	118 (63)	129 (67)	122 (68)	.34

Abbreviations: BMI, body mass index (calculated as weight in kilograms divided by height in meters squared); eGFR, estimated glomerular filtration rate; MFI, mean fluorescence intensity; TIA, transient ischemic attack.

^a^ Data are presented as number (percentage) of participants unless otherwise indicated. Participants were classified as having any systemic symptoms (fever, chills, muscle pain, nausea, vomiting, headache, and/or moderate to severe fatigue), local symptoms only (injection site pain and/or rash), or no symptoms after either messenger RNA vaccine dose.

^b^ *P* values are from the χ^2^ test or unpaired *t* test.

^c^ Other racial or ethnic group includes American Indian or Alaska Native, Asian, Black, and Hispanic or Latino.

**Figure.**
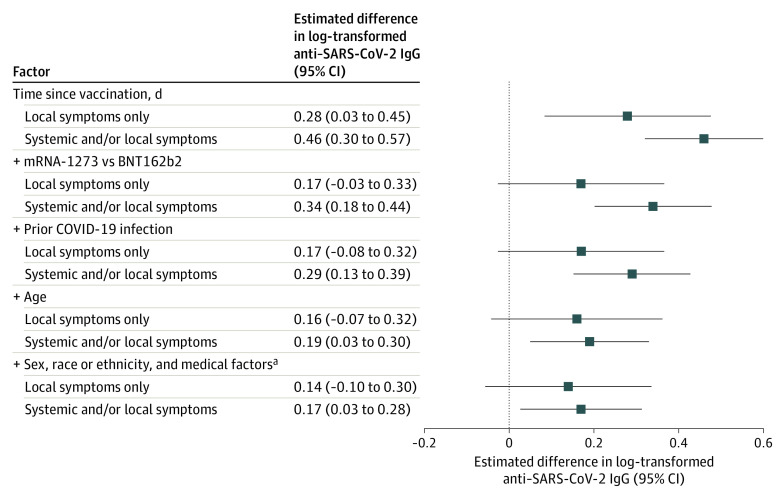
Association of Self-reported Symptoms After SARS-CoV-2 Messenger RNA Vaccination With Continuous Log-Transformed Values for Anti-Spike IgG Antibodies Among 928 Fully Vaccinated Framingham Heart Study Participants, February 2021 to January 2022 Effect estimates for self-reported symptoms compared with no symptoms with 95% CIs. Plus signs indicate that those factors were sequentially added to the model. ^a^Medical factors include body mass index, smoking status, diabetes, hypertension, coronary heart disease, heart failure, stroke or transient ischemic attack, and estimated glomerular filtration rate.

## Discussion

In a sample of twice-vaccinated, older, community-dwelling US adults, self-reported systemic symptoms after SARS-CoV-2 mRNA vaccination were associated with greater antibody response vs local-only or no symptoms. These results agree with a previous report^6^ in US health care workers that showed higher postvaccination antibody measurements among those with significant symptoms after an mRNA vaccine. This report identifies age, sex, and Moderna vaccine as factors associated with both vaccine reactogenicity and immunogenicity, consistent with prior observations.^3,6^ No association was observed between symptoms after vaccination and race or ethnicity, body mass index, or comorbidities. In this generalizable cohort, nearly all participants exhibited a positive antibody response to complete mRNA vaccine series. Nonetheless, systemic symptoms remained associated with greater antibody response in multivariable-adjusted models, highlighting unexplained interpersonal variability. Further research on biological mechanisms underlying heterogeneity in vaccine response is needed. Limitations of this report include an older, predominantly non-Hispanic White, professional cohort; potential recall bias; and use of MFI, which is not standardized against neutralizing antibody titers. In conclusion, these findings support reframing postvaccination symptoms as signals of vaccine effectiveness and reinforce guidelines for vaccine boosters in older adults.

## Notes

Supplement. **eFigure.** Flowchart of Study Participants Who Completed the C4R Questionnaire, Submitted a Dried Blood Spot for Evaluation and Received Two Doses of Either Pfizer-Biontech or Moderna SARS Cov-2 Vaccines
