# Error in Figure and Discussion

**DOI:** 10.1001/jamanetworkopen.2022.47057

**Published:** 2022-11-28

In the Research Letter titled “Association of Symptoms After COVID-19 Vaccination With Anti–SARS-CoV-2 Antibody Response in the Framingham Heart Study,”^1^ published October 21, 2022, the Figure has been corrected to change some of the row headers to “Systemic with or without local symptoms” where they previously said “Systemic and/or local symptoms”; also, the first sentence of the Discussion has been corrected to include missing details on attenuation of the reported associations. This article has been corrected.^1^
