# Supplementary material for: Association of Symptoms After COVID-19 Vaccination With Anti–SARS-CoV-2 Antibody Response in the Framingham Heart Study
Source: JAMA Netw Open. 2022 Oct 21;5(10):e2237908. doi: 10.1001/jamanetworkopen.2022.37908 (PMC9587476; doi:10.1001/jamanetworkopen.2022.37908)
Supplement: Supplement. — eFigure. Flowchart of Study Participants Who Completed the C4R Questionnaire, Submitted a Dried Blood Spot for Evaluation and Received Two Doses of Either Pfizer-Biontech or Moderna SARS Cov-2 Vaccines [file jamanetwopen-e2237908-s001.pdf]

Supplementary Online Content

Hermann EA, Lee B, Balte PP, et al. Association of symptoms after COVID-19 vaccination with anti–SARS-CoV-2 antibody response in the Framingham Heart Study. *JAMA Netw Open*. 2022;5(10):e2237908. doi:10.1001/jamanetworkopen.2022.37908

eFigure. Flowchart of Study Participants Who Completed the C4R Questionnaire, Submitted a Dried Blood Spot for Evaluation and Received Two Doses of Either Pfizer-Biontech or Moderna SARS Cov-2 Vaccines

This supplementary material has been provided by the authors to give readers additional information about their work.

© 2022 Hermann EA et al. *JAMA Network Open.*

eFigure. Flowchart of Study Participants Who Completed the C4R Questionnaire, Submitted a Dried Blood Spot for Evaluation and Received Two Doses of Either Pfizer-Biontech or Moderna SARS Cov-2 Vaccines

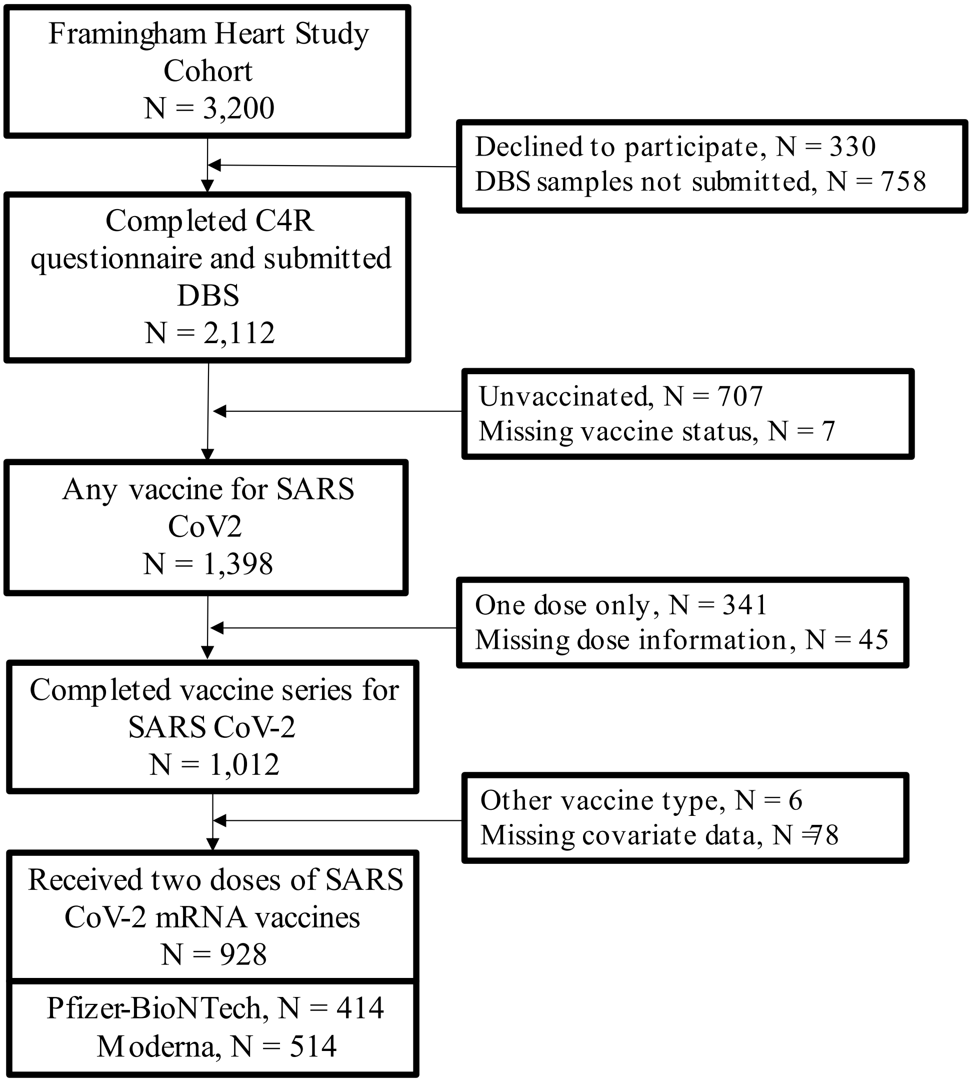

© 2022 Hermann EA et al. *JAMA Network Open.*
